# Supplementary material for: Mechanism of millisecond Lys48-linked poly-ubiquitin chain formation by cullin-RING ligases
Source: Nat Struct Mol Biol. 2024 Feb 7;31(2):378–89. doi: 10.1038/s41594-023-01206-1 (PMC10873206; doi:10.1038/s41594-023-01206-1)
Supplement: Supplementary file 1 — Supplementary Fig. 1. [file 41594_2023_1206_MOESM1_ESM.pdf]

nature structural & molecular biology

Article https://doi.org/10.1038/s41594-023-01206-1

Mechanism of millisecond Lys48-linked poly-ubiquitin chain formation by cullin-RING ligases

In the format provided by the authors and unedited

EMD-17803; EMD-17822; PDB 8PQL

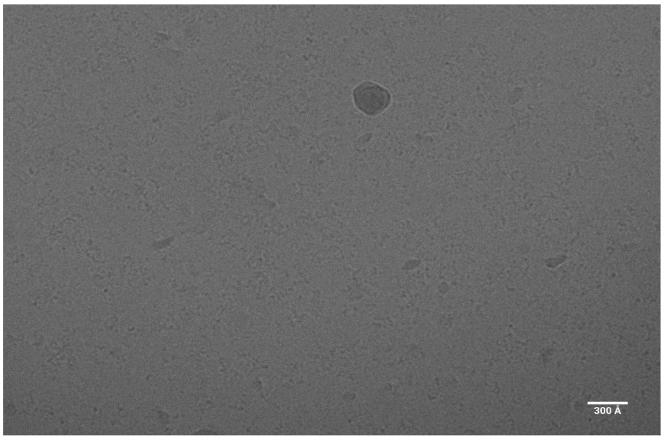

EMD-17798; EMD-17799

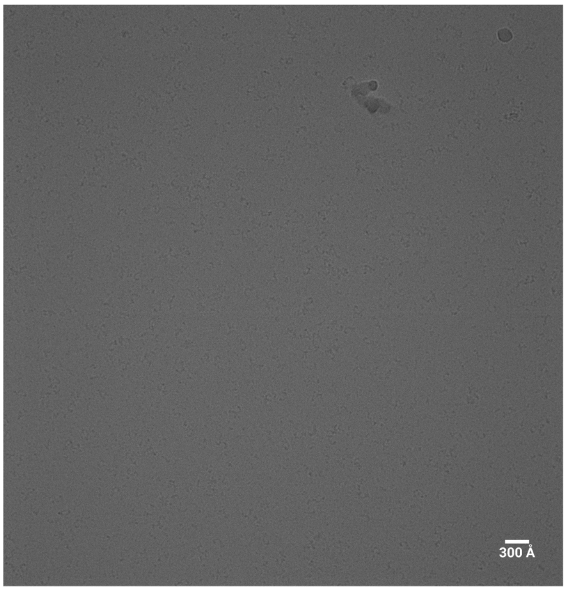

EMD-18767

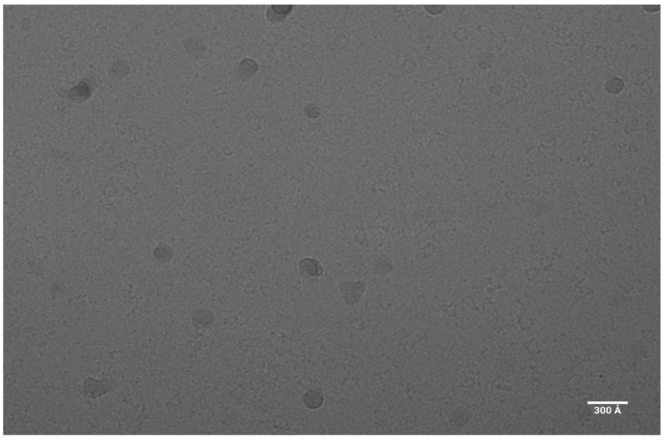

EMD-17800; EMD-17801

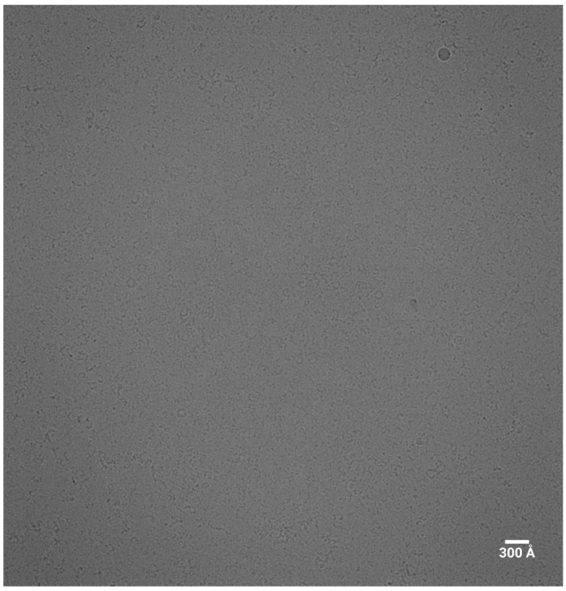

EMD-17802

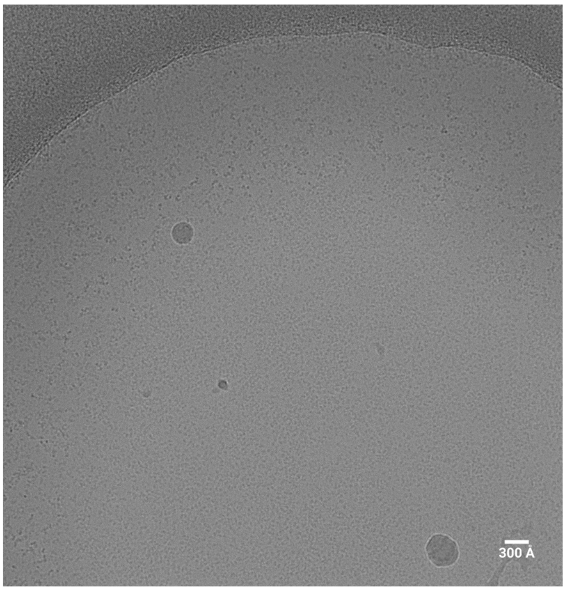

Supplementary Figure 1. Representative micrographs showing individual particles for the various CRL complexes reported in this study. EMD-17803 and EMD-17822 correspond to the neddylated $CRL2^{FEM1C}$ poly-ubiquitin chain formation complex with UBE2R2~ubiquitin and Sil1-ubiquitin substrate. EMD-18767 corresponds to the neddylated $CRL2^{VHL-MZ1-BRD4}$ poly-ubiquitin chain formation complex with UBE2R2~ubiquitin, the PROTAC MZ1, and the neo-substrate fragment C356A C357A C391A C429A BRD4 (residues 352-460) fused to ubiquitin. EMD-17802 corresponds to the neddylated $CRL1^{FBXW7}$ poly-ubiquitin chain formation complex with UBE2R2~ubiquitin and Cyclin E-ubiquitin substrate. EMD-17798 and EMD-17799 correspond to the unneddylated $CRL2^{FEM1C}$ complex, and EMD-17800 and EMD-17801 to the neddylated $CRL2^{FEM1C}$ complex.
