# Supplementary material for: Mechanism of millisecond Lys48-linked poly-ubiquitin chain formation by cullin-RING ligases
Source: Nat Struct Mol Biol. 2024 Feb 7;31(2):378–89. doi: 10.1038/s41594-023-01206-1 (PMC10873206; doi:10.1038/s41594-023-01206-1)
Supplement: Supplementary file 2 — Reporting Summary [file 41594_2023_1206_MOESM2_ESM.pdf]

nature portfolio

Corresponding author(s): Brenda A. Schulman
Gary Kleiger

Last updated by author(s): 12/14/2023

Reporting Summary

Nature Portfolio wishes to improve the reproducibility of the work that we publish. This form provides structure for consistency and transparency in reporting. For further information on Nature Portfolio policies, see our Editorial Policies and the Editorial Policy Checklist.

Statistics

For all statistical analyses, confirm that the following items are present in the figure legend, table legend, main text, or Methods section.

n/a	Confirmed	
☐	☒	The exact sample size (*n*) for each experimental group/condition, given as a discrete number and unit of measurement
☐	☒	A statement on whether measurements were taken from distinct samples or whether the same sample was measured repeatedly
☒	☐	The statistical test(s) used AND whether they are one- or two-sided<br>*Only common tests should be described solely by name; describe more complex techniques in the Methods section.*
☒	☐	A description of all covariates tested
☒	☐	A description of any assumptions or corrections, such as tests of normality and adjustment for multiple comparisons
☐	☒	A full description of the statistical parameters including central tendency (e.g. means) or other basic estimates (e.g. regression coefficient) AND variation (e.g. standard deviation) or associated estimates of uncertainty (e.g. confidence intervals)
☒	☐	For null hypothesis testing, the test statistic (e.g. *F*, *t*, *r*) with confidence intervals, effect sizes, degrees of freedom and *P* value noted<br>*Give P values as exact values whenever suitable.*
☒	☐	For Bayesian analysis, information on the choice of priors and Markov chain Monte Carlo settings
☒	☐	For hierarchical and complex designs, identification of the appropriate level for tests and full reporting of outcomes
☒	☐	Estimates of effect sizes (e.g. Cohen's *d*, Pearson's *r*), indicating how they were calculated

Our web collection on statistics for biologists contains articles on many of the points above.

Software and code

Policy information about availability of computer code

Data collection	Cryo-EM: SerialEM v3.8.0-b5. Gel imaging: Amersham Imager 600, Amersham Typhoon
Data analysis	Cryo-EM: RELION v3.1.1, MotionCorr2 v. 1.1.0, CryoSparc v4.2.0, CTFFIND v4.1, and Gautomatch v0.56. Structure Analysis and Visualization: Chimera v1.15 and ChimeraX v1.4. Model Building: COOT v0.9.6, Phenix.refine v1.19.2-4158, DeepEMhancer (https://github.com/rsanchezgarc/deepEMhancer), and AlphaFold2. Crosslinking mass spectrometry: Proteome Discoverer v2.5.0.400. Biochemistry: Prism v9 (Graphpad), Mathematica v13.1 (Wolfram), ImageQuant v8.2.0.0 (Cytiva).

For manuscripts utilizing custom algorithms or software that are central to the research but not yet described in published literature, software must be made available to editors and reviewers. We strongly encourage code deposition in a community repository (e.g. GitHub). See the Nature Portfolio guidelines for submitting code & software for further information.

Data

Policy information about availability of data

All manuscripts must include a data availability statement. This statement should provide the following information, where applicable:

- Accession codes, unique identifiers, or web links for publicly available datasets
- A description of any restrictions on data availability
- For clinical datasets or third party data, please ensure that the statement adheres to our policy

The atomic coordinates and electron microscopy maps have been deposited in the PDB with accession code 8PQL and in the Electron Microscopy Data Bank with codes EMD-17803 (consensus map) and EMD-17822 (composite map) for the neddylated CRL2FEM1C poly-ubiquitin chain formation complex, EMD-17802 for the neddylated CRL1FBXW7 poly-ubiquitin chain formation complex, EMD-18767 for the neddylated CRL2VHL-MZ1-BRD4 poly-ubiquitin chain formation complex, EMD-17798 and EMD-17799 for the CRL2FEM1C complex, and EMD-17800 and EMD-17801 for the neddylated CRL2FEM1C complex. Publicly available PDB entries are 1LDJ, 1LM8, 4AP4, 5AIT, 5N4W, 6LBN, 6NYO, 6TTU, 7B5L, 7MEY, and 7OJX. Source data are provided with this paper.

Research involving human participants, their data, or biological material

Policy information about studies with human participants or human data. See also policy information about sex, gender (identity/presentation), and sexual orientation and race, ethnicity and racism.

Reporting on sex and gender	No research involving human participants has been performed
Reporting on race, ethnicity, or other socially relevant groupings	No research involving human participants has been performed
Population characteristics	No research involving human participants has been performed
Recruitment	No research involving human participants has been performed
Ethics oversight	No research involving human participants has been performed

Note that full information on the approval of the study protocol must also be provided in the manuscript.

Field-specific reporting

Please select the one below that is the best fit for your research. If you are not sure, read the appropriate sections before making your selection.

☒ Life sciences ☐ Behavioural & social sciences ☐ Ecological, evolutionary & environmental sciences

For a reference copy of the document with all sections, see nature.com/documents/nr-reporting-summary-flat.pdf

Life sciences study design

All studies must disclose on these points even when the disclosure is negative.

Sample size	N≥2. Sample sizes were chosen such that SEMs are typically 10% of the estimated values (and no more than 25%).
Data exclusions	no exclusions were made
Replication	N≥2 All experiments were performed independently. All attempts at replication were successful.
Randomization	Samples are not grouped as all comparisons are pairwise.
Blinding	Samples are not grouped such that blinding is not applicable to the study.

Reporting for specific materials, systems and methods

We require information from authors about some types of materials, experimental systems and methods used in many studies. Here, indicate whether each material, system or method listed is relevant to your study. If you are not sure if a list item applies to your research, read the appropriate section before selecting a response.

Materials & experimental systems

n/a	Involved in the study	
☒	☐	Antibodies
☐	☒	Eukaryotic cell lines
☒	☐	Palaeontology and archaeology
☒	☐	Animals and other organisms
☒	☐	Clinical data
☒	☐	Dual use research of concern
☒	☐	Plants

Methods

n/a	Involved in the study	
☒	☐	ChIP-seq
☒	☐	Flow cytometry
☒	☐	MRI-based neuroimaging

Eukaryotic cell lines

Policy information about cell lines and Sex and Gender in Research

Cell line source(s)	High five cell (BTI-TN-5B1-4) were obtained from ThermoFisher Scientific (catalogue number:B85502). Gibco Sf9 cells were obtained ThermoFisher Scientific (catalogue number:11496016).
Authentication	Cell lines were not authenticated.
Mycoplasma contamination	Cell lines were tested regularly for mycoplasma with no contamination detected.
Commonly misidentified lines (See ICLAC register)	No commonly misidentified cell lines were used in this study.
