# Supplementary figures and images for: Mechanism of millisecond Lys48-linked poly-ubiquitin chain formation by cullin-RING ligases
Source: Nat Struct Mol Biol. 2024 Feb 7;31(2):378–89. doi: 10.1038/s41594-023-01206-1 (PMC10873206; doi:10.1038/s41594-023-01206-1)

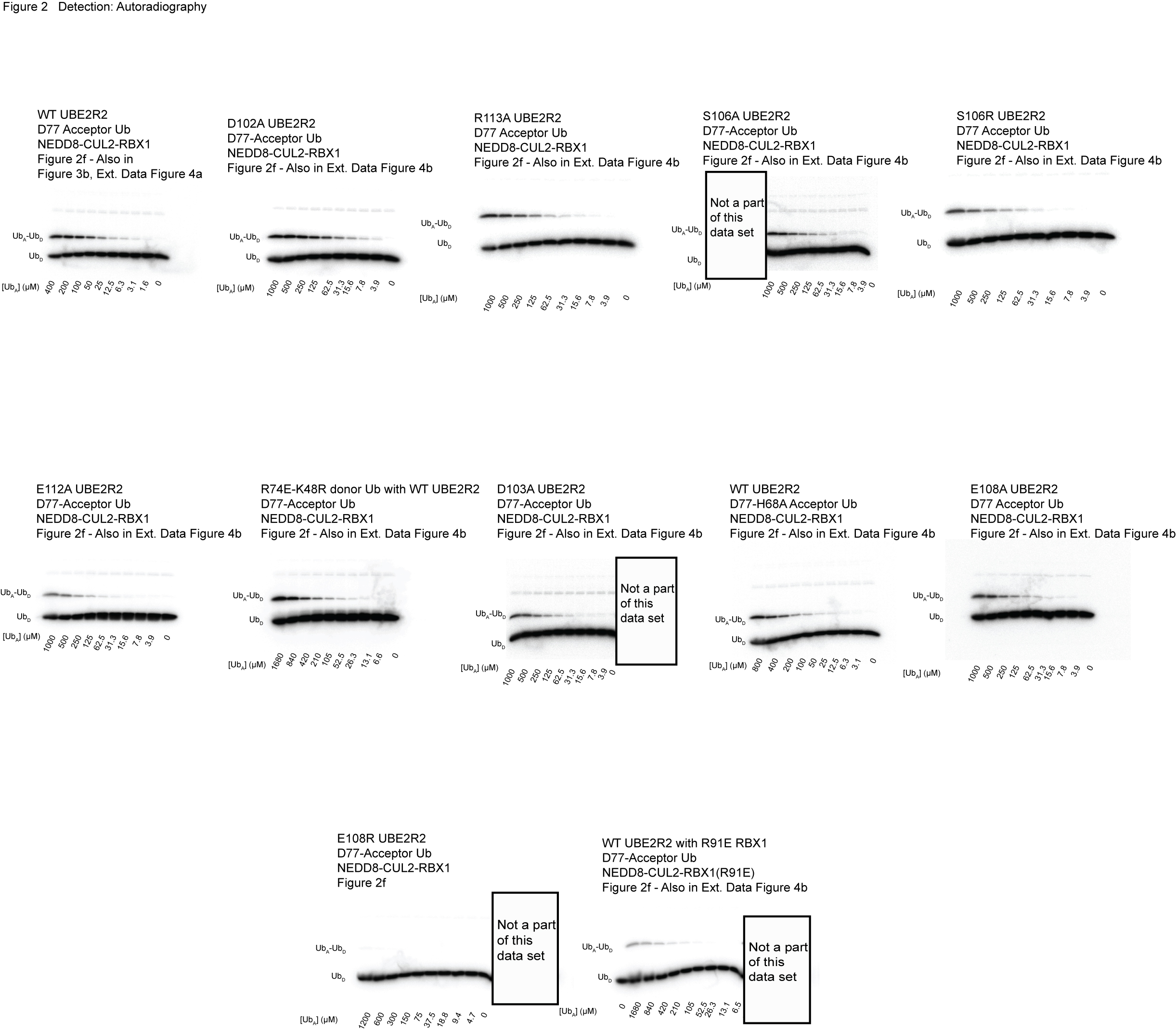

Supplement: Supplementary file 8 — Unprocessed autoradiograms. [file 41594_2023_1206_MOESM8_ESM.jpg]

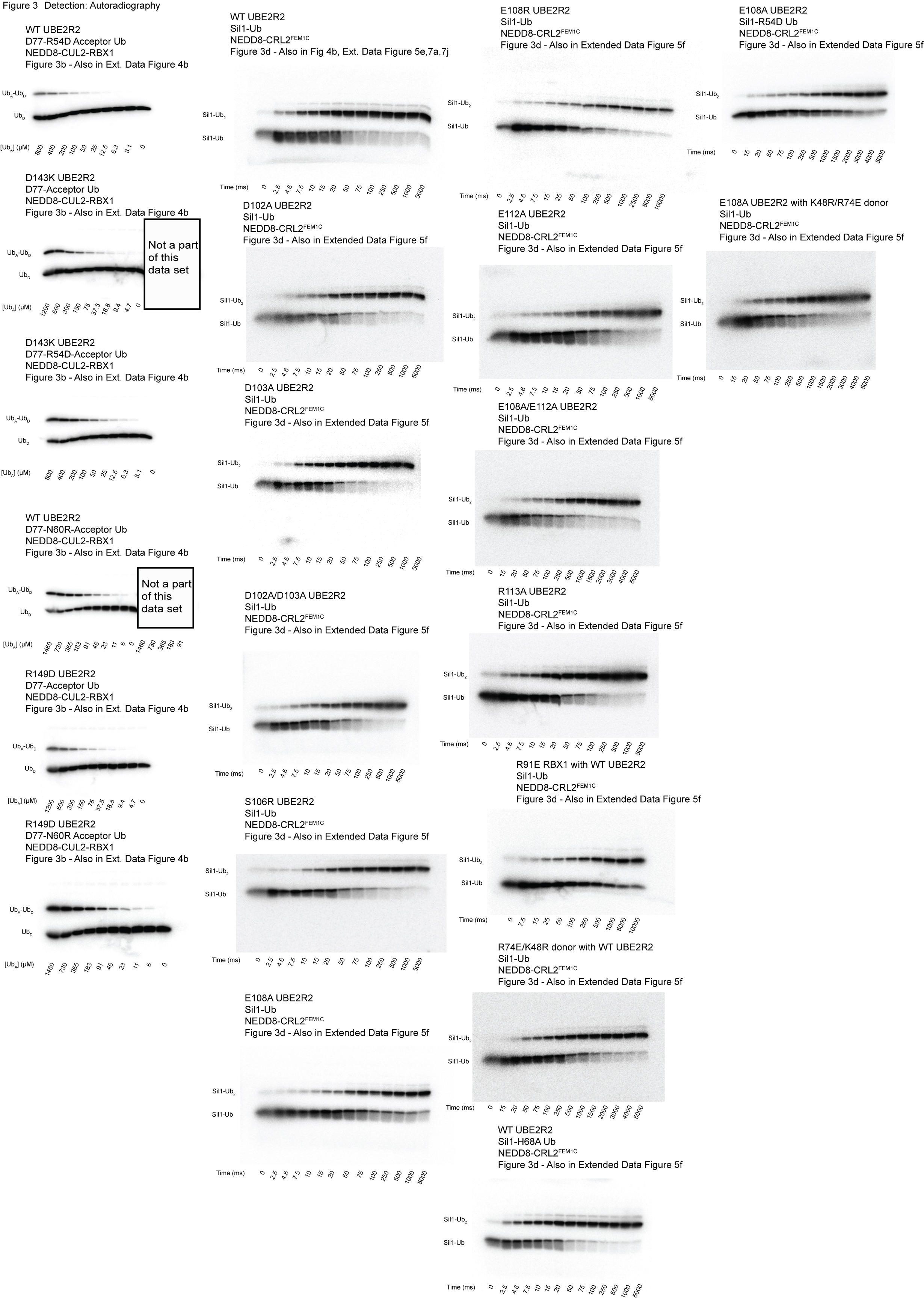

Supplement: Supplementary file 10 — Unprocessed autoradiograms. [file 41594_2023_1206_MOESM10_ESM.jpg]

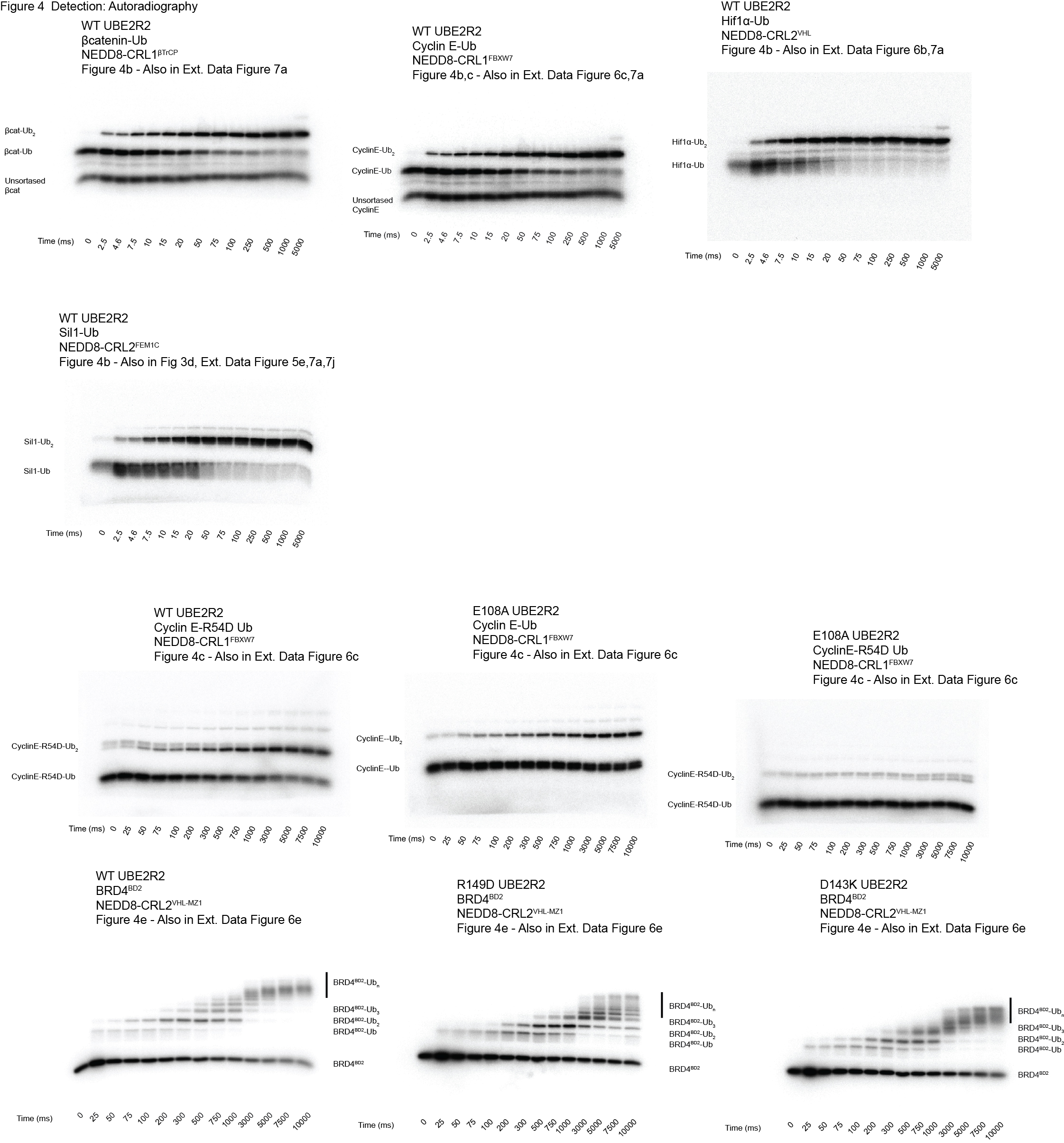

Supplement: Supplementary file 12 — Unprocessed autoradiograms. [file 41594_2023_1206_MOESM12_ESM.jpg]

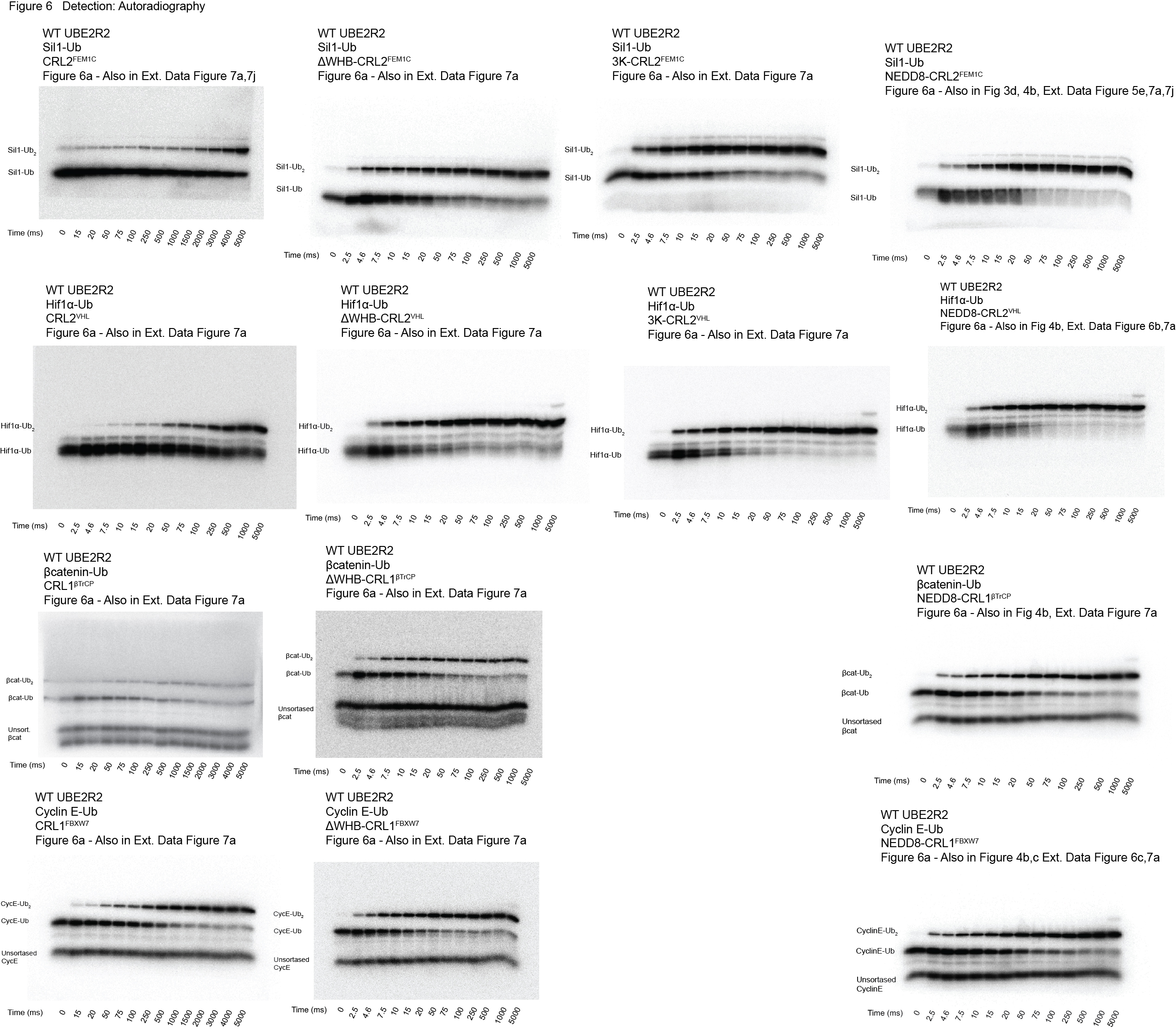

Supplement: Supplementary file 14 — Unprocessed autoradiograms. [file 41594_2023_1206_MOESM14_ESM.jpg]

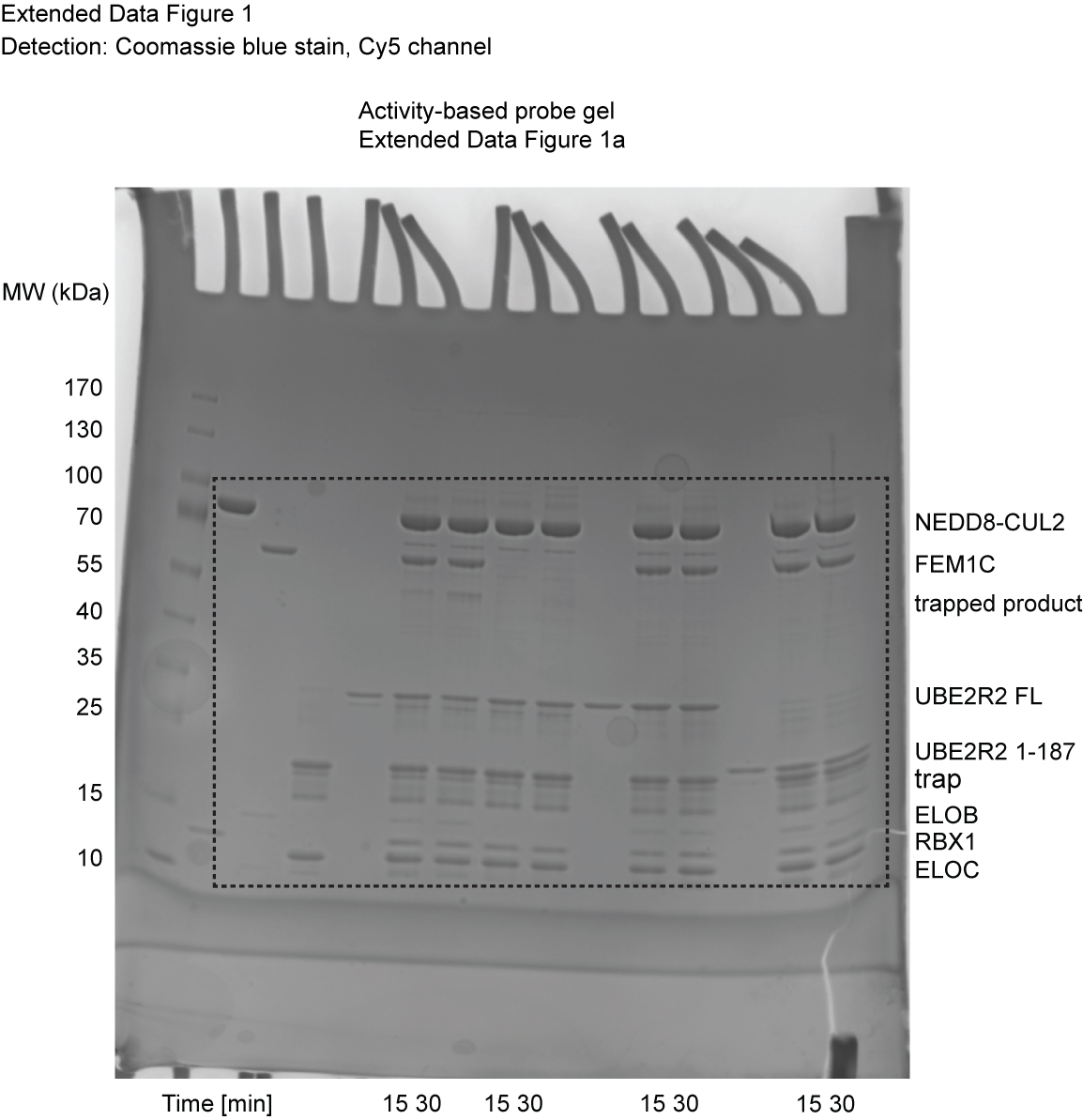

Supplement: Supplementary file 16 — Unprocessed Coomassie-stained SDS–PAGE gel. [file 41594_2023_1206_MOESM16_ESM.jpg]

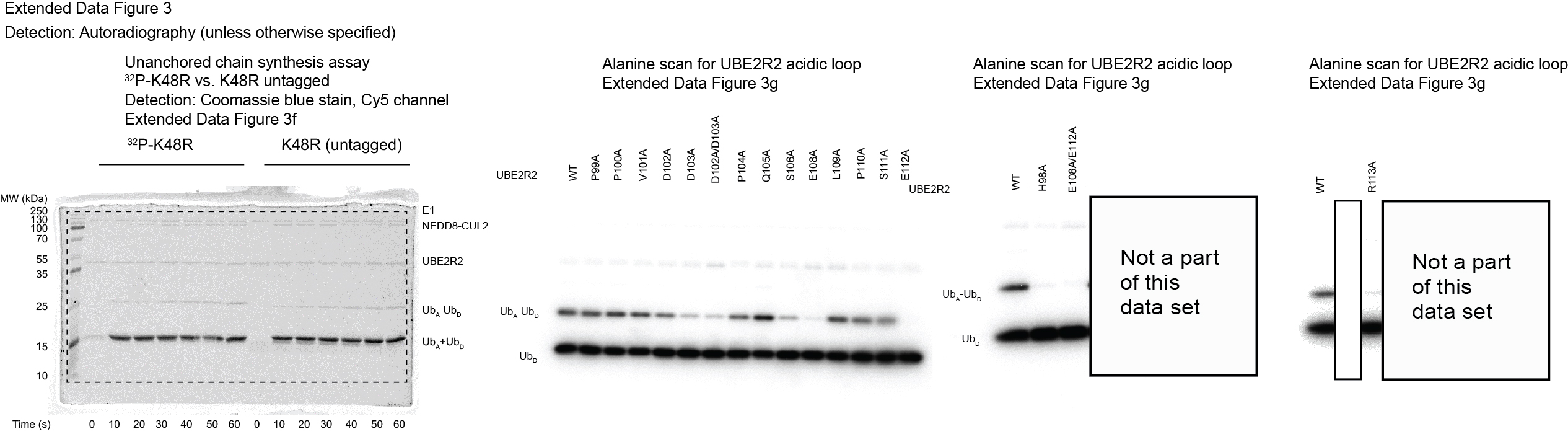

Supplement: Supplementary file 17 — Unprocessed Coomassie-stained SDS–PAGE gel and autoradiograms. [file 41594_2023_1206_MOESM17_ESM.jpg]

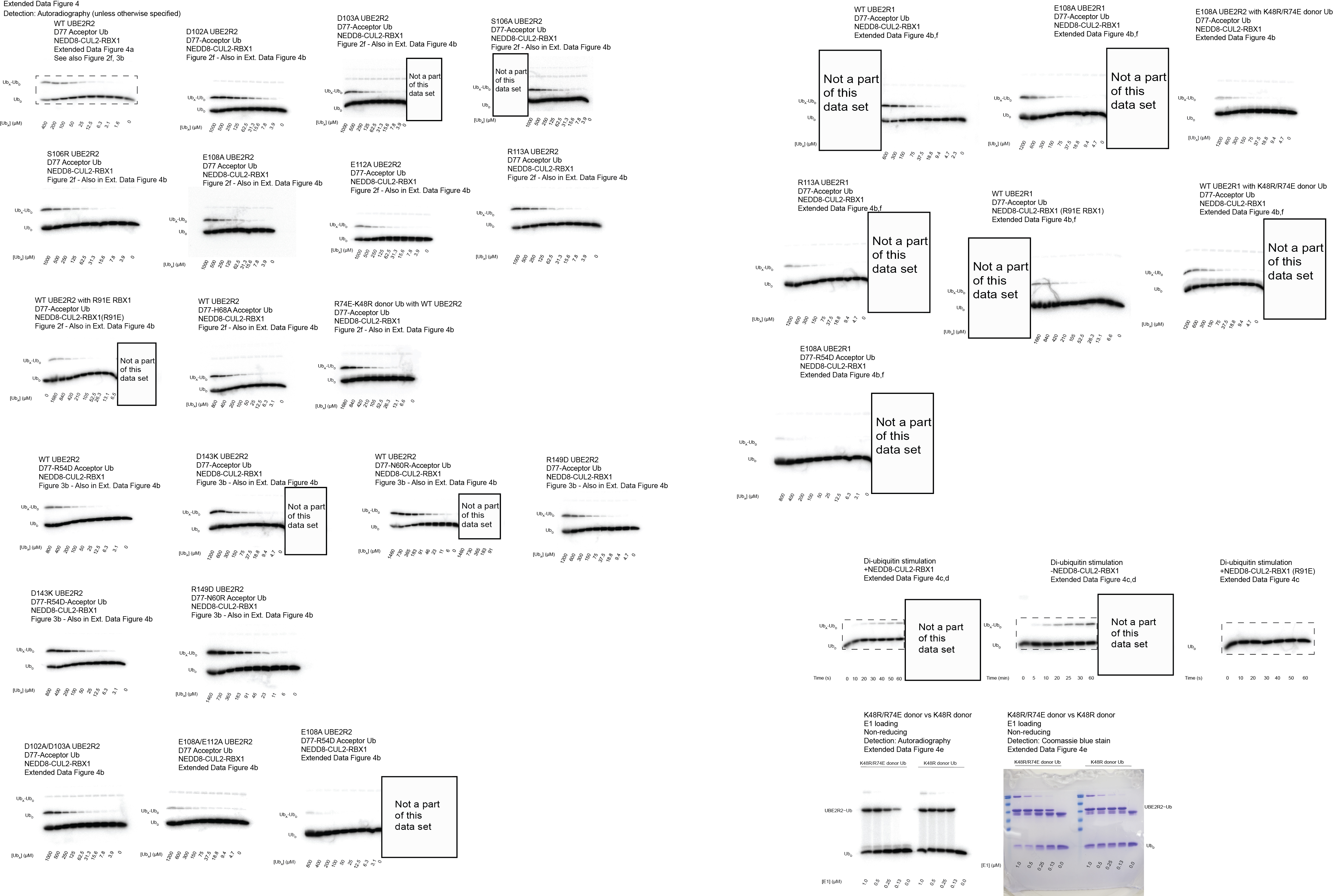

Supplement: Supplementary file 19 — Unprocessed Coomassie-stained SDS–PAGE gel and autoradiograms. [file 41594_2023_1206_MOESM19_ESM.jpg]

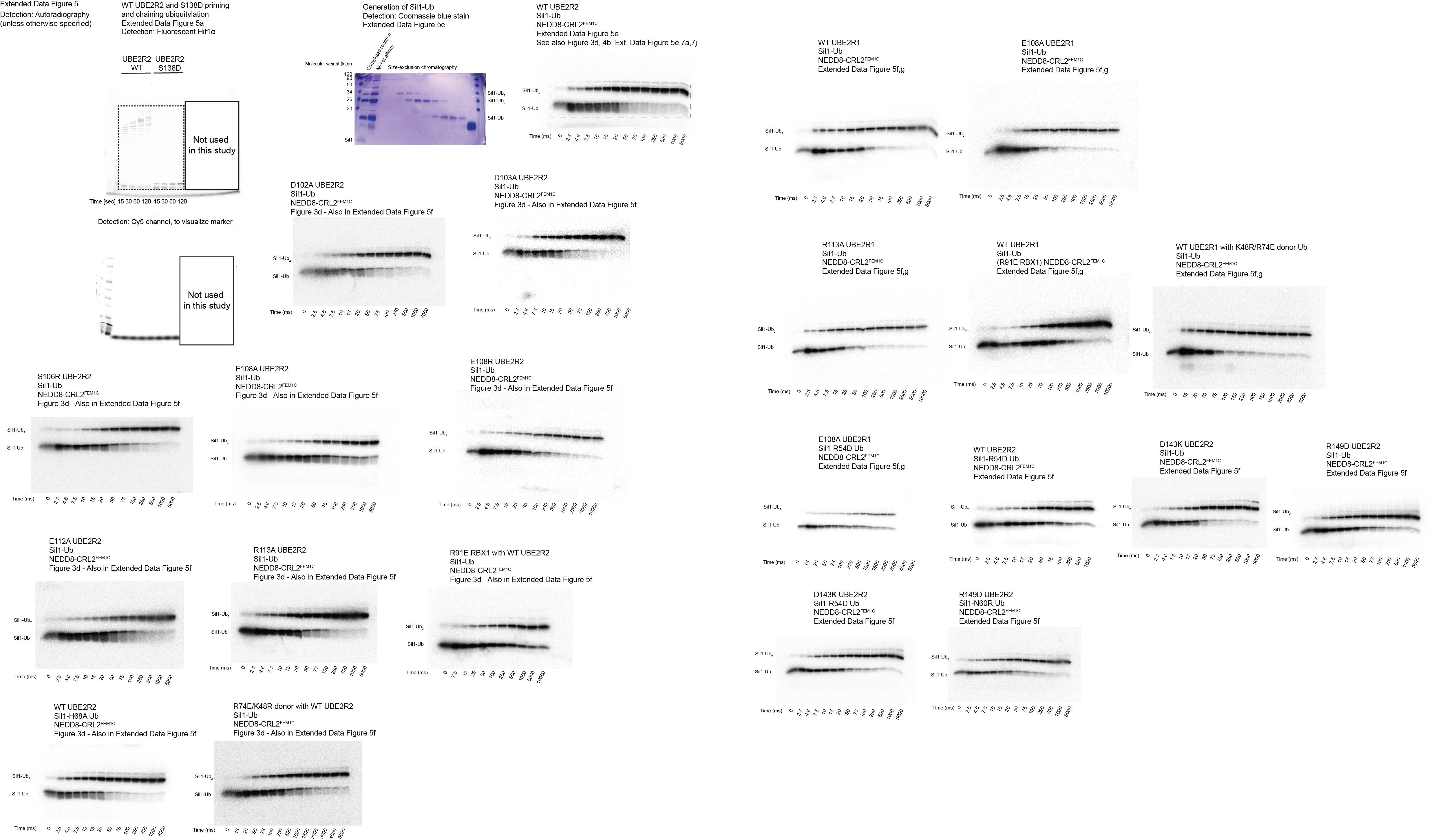

Supplement: Supplementary file 21 — Unprocessed Coomassie-stained SDS–PAGE gel, fluorescence-scanned gel and autoradiograms. [file 41594_2023_1206_MOESM21_ESM.jpg]

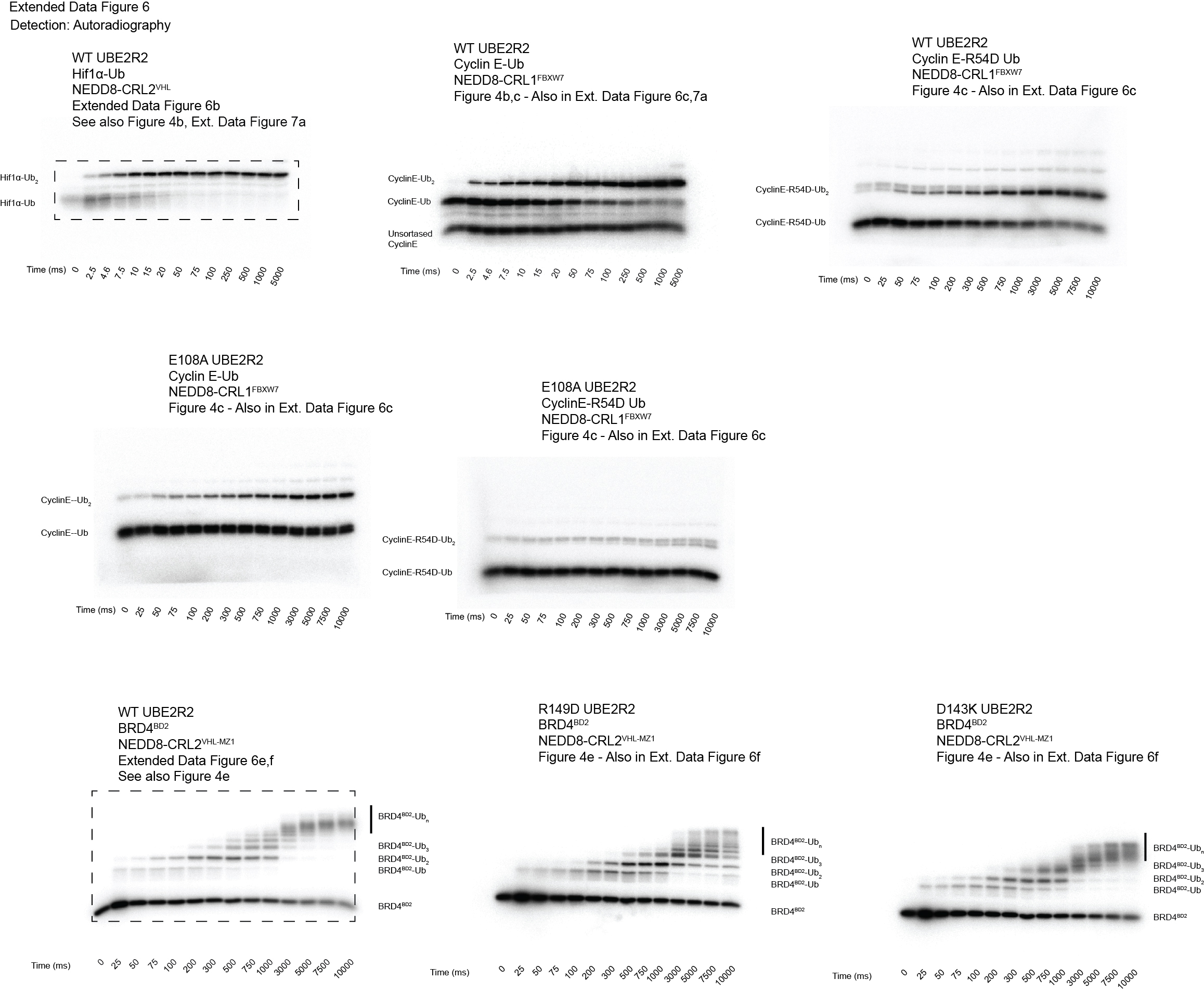

Supplement: Supplementary file 23 — Unprocessed autoradiograms. [file 41594_2023_1206_MOESM23_ESM.jpg]

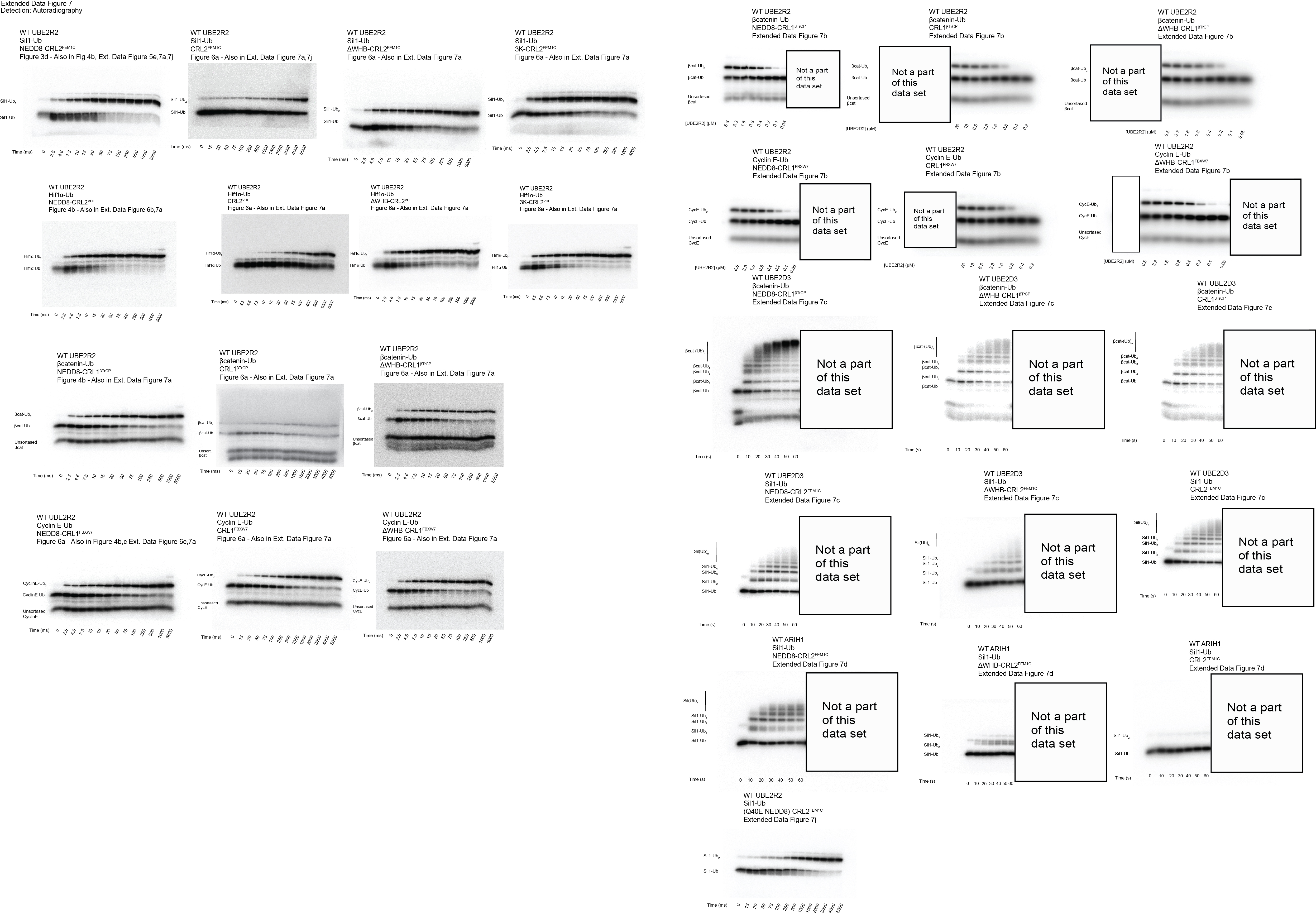

Supplement: Supplementary file 25 — Unprocessed autoradiograms. [file 41594_2023_1206_MOESM25_ESM.jpg]
